# Supplementary material for: Performance of patient acuity rating by rapid response team nurses for predicting short-term prognosis
Source: PLoS One. 2019 Nov 14;14(11):e0225229. doi: 10.1371/journal.pone.0225229 (PMC6855430; doi:10.1371/journal.pone.0225229)
Supplement: S1 Table — (DOCX) [file pone.0225229.s001.docx]

**S1 Table. Calculation of Modified Early Warning Score**

| Category | 3 | 2 | 1 | 0 | 1 | 2 | 3 |
| --- | --- | --- | --- | --- | --- | --- | --- |
| Respiratory rate (bpm) |  | <9 |  | 9–14 | 15–20 | 21–29 | ≥30 |
| Heart rate (bpm) |  | <40 | 41–50 | 51–100 | 101–110 | 111–129 | ≥130 |
| Systolic blood pressure (mmHg) | <70 | 71–80 | 81–100 | 101–199 |  | ≥200 |  |
| Temperature (℃) |  | <35 |  | 35–38.4 |  | ≥38.5 |  |
| AVPU score |  |  |  | Alert | Reacting to Voice | Reacting to Pain | Unresponsive |
