# Supplementary material for: Performance of patient acuity rating by rapid response team nurses for predicting short-term prognosis
Source: PLoS One. 2019 Nov 14;14(11):e0225229. doi: 10.1371/journal.pone.0225229 (PMC6855430; doi:10.1371/journal.pone.0225229)
Supplement: S2 Table — (DOCX) [file pone.0225229.s002.docx]

**S2 Table. Calculation of VitalPAC Early Warning Score**

| Category | 3 | 2 | 1 | 0 | 1 | 2 | 3 |
| --- | --- | --- | --- | --- | --- | --- | --- |
| Respiratory rate (bpm) | ≤8 |  | 9–11 | 12–20 |  | 21–24 | ≥25 |
| SaO_2_ (%) | ≤91 | 92–93 | 94–95 | ≥96 |  |  |  |
| Inspired O_2_ |  |  |  | Air |  |  | Any O_2_ |
| Heart rate (bpm) |  | ≤40 | 41–50 | 51–90 | 91–110 | 111–130 | ≥131 |
| Systolic blood pressure (mmHg) | ≤90 | 91–100 | 101–110 | 111–249 | ≥250 |  |  |
| Temperature (℃) | ≤35.0 |  | 35.1–36.0 | 36.1–38.0 | 38.1–39.0 | ≥39.1 |  |
| AVPU score |  |  |  | Alert |  |  | Reacting to voice, pain, or unresponsive |
