# Supplementary material for: Performance of patient acuity rating by rapid response team nurses for predicting short-term prognosis
Source: PLoS One. 2019 Nov 14;14(11):e0225229. doi: 10.1371/journal.pone.0225229 (PMC6855430; doi:10.1371/journal.pone.0225229)
Supplement: S3 Table — (DOCX) [file pone.0225229.s003.docx]

**S3 Table. Calculation of Standardised Early Warning Score**

| Category | 3 | 2 | 1 | 0 | 1 | 2 | 3 |
| --- | --- | --- | --- | --- | --- | --- | --- |
| Respiratory rate (bpm) | ≤8 |  |  | 9–20 | 21–30 | 31–35 | ≥36 |
| SaO_2_ (%) | <85 | 85–89 | 90–92 | ≥93 |  |  |  |
| Heart rate (bpm) | ≤29 | 30–39 | 40–49 | 50–99 | 100–109 | 110–129 | ≥130 |
| Blood pressure (mmHg) | ≤69 | 70–79 | 80–89 | 100–199 |  | ≥200 |  |
| Temperature (℃) | ≤33.9 | 34–34.9 | 35–35.9 | 36–37.9 | 38–38.9 | ≥39 |  |
| AVPU score |  |  |  | Alert | Reacting to Voice | Reacting to Pain | Unresponsive |
