# Supplementary material for: Performance of patient acuity rating by rapid response team nurses for predicting short-term prognosis
Source: PLoS One. 2019 Nov 14;14(11):e0225229. doi: 10.1371/journal.pone.0225229 (PMC6855430; doi:10.1371/journal.pone.0225229)
Supplement: S4 Table — (DOCX) [file pone.0225229.s004.docx]

**S4 Table. Calculation of Cardiac Arrest Risk Triage**

| Category | 0 | 4 | 6 | 8 | 9 | 12 | 13 | 15 | 22 |
| --- | --- | --- | --- | --- | --- | --- | --- | --- | --- |
| Respiratory rate (bpm) | <21 |  |  | 21–23 |  | 24–25 |  | 26–29 | >29 |
| Heart rate (bpm) | <110 | 110–139 |  |  |  |  | >139 |  |  |
| Diastolic blood pressure (mmHg) | >49 | 40–49 | 35–39 |  |  |  | <35 |  |  |
| Age | <55 | 55–69 |  |  | >69 |  |  |  |  |
