# Supplementary material for: Performance of patient acuity rating by rapid response team nurses for predicting short-term prognosis
Source: PLoS One. 2019 Nov 14;14(11):e0225229. doi: 10.1371/journal.pone.0225229 (PMC6855430; doi:10.1371/journal.pone.0225229)
Supplement: Attachment — Submitted filename: report plos 1.19.docx [file pone.0225229.s006.docx]

Reviewer's report (1): 
REVIEW: Yeon Lee et al., 
General comment: This is an interesting study evaluating a score with good scientific hypothesis which is Patient Acuity Rating by Nurses in Rapid Response Team. The study is important as a clinical tool and would add to the decision making in the subjected group which is people suffering cardiac arrest.  

Title:

“Patient Acuity Rating by Nurses in Rapid Response Team”. The title is inappropriate; I think it would be better to address what you intend to do in the study which is score evaluation in the population you made because with this title it looks like a review article e.g evaluation, assessment

1) Abstract: Not acceptable in the current format , the abstract is poorly written, there is no background. I suggest the following to improve the abstract

- Background , the background should not describe the score the authors intend to study, but it should include literatures to support the hypothesis
- Aim of the work is not clear
- Methods : the authors should not include the statement “is that this work is based on a retrospective review of a prospective observational cohort” This make the reader loss interest indeed they should include methodological summary, a short description of the acuity score, if they used additional instruments, I think the study intend to test two scores which should come here, sample volume type of the study should come in the methodology, the study subjects, should include the type of sampling.
- The population of the study is not described whether they are nurse or nurses and physicians, I think this critical , the authors focused on the patient’s outcome however there is a factor related to the observer , so the observer data should be described and included in the statistical analysis
- Results: the results should start with descriptive numbers from the study population followed by the rest, the authors did not use abbreviation for the score also it is mentioned many times, I do not know why they use capital letters in multiple occasions.
- Conclusions: The conclusion should summarize your results in a better attractive way
- I think that abstract needs to be revised by a professional English editor

2) Introduction:

- The introduction suffers a lot of redundancy, it should be more focused.
- The introduction should include the background of the importance of decisions by the rapid response team,
- The score doe not needs to be described in this section but its importance in different population should be highlighted

3) Hypothesis:

- The authors highlighted that they intend to “This study aimed to evaluate the accuracy of the PAR compared to other early warning scores when scored by RRT” the other scoring system needs to be mentioned like A vesrus B

4) Methodology 
a. The methodology as the rest of the manuscript suffers a critical lack of focusing issues

b. The authors do not need to mention that this study is based on the others work, they should cite to this score in the introduction and start the methodology by what they did, the work is considered original if it is conducted in another population and or different sample volume.  
c. a figure including the score they used is needed for the reader “available at”

J Hosp Med. 2011 October ; 6(8): 475–479. doi:10.1002/jhm.886.

- d. The population of the study is not clearly described whether they are nurse or nurses and physicians, I think this critical , the authors focused on the patient’s outcome however there is a factor related to the observer diversity, so the observer data should be described and included in the statistical analysis. The number also is essential , the demographics within the group is important e.g within the analyzing nurses what is the total years of experience, whether they share the same precision of assessment

e. *Ethical Considerations:*

Participant identity kept confidential, final report would not contain any identity. Comprehensive explanation for the participants about the questionnaires, the type, purpose of the study and outcome was done, early rejection, or late withdrawal was permissive. Ethical approval was obtained according to the corporate regulation

f. reliability and validity score for the used questionnaires had not been identified this needs to be checked by a statistician, which include a secondary analysis related to the observers

5) Results 
a. The results should be revised with new analysis including the observers data as requested
6) Statistical review: the manuscript needs to be seen by a statistician

the study outcome section needs to incorporated into the statistical analysis

7) Discussion: 
a. I think the manuscript needs major revision by the authors to decide upon how the discussion will go 
b. The discussion is too long and it should start with the salient findings followed by focused analysis

c. The discussion needs 2^nd^ round of revision after fixing the earlier issues

8) Level of interest: An article of importance in its field 

9) Quality of written English: Need secondary revision 
10) Ethical concerns: The author explained that they obtained waived ethical approval from their institute regarding the patients but they did should include approval related to the participation of nurses

11) References: 
a. The references style was not adequately followed according to Plos One style. 
12) Declaration of competing interests: I declare that I have no competing interests.
