# Supplementary material for: Performance of patient acuity rating by rapid response team nurses for predicting short-term prognosis
Source: PLoS One. 2019 Nov 14;14(11):e0225229. doi: 10.1371/journal.pone.0225229 (PMC6855430; doi:10.1371/journal.pone.0225229)
Supplement: Attachment — Submitted filename: 1_Response_to_editor_and_reviewer_revised.docx [file pone.0225229.s007.docx]

**Dear editor:**

We would like to thank all the editors and reviewers for providing us with their valuable opinions about our manuscript. We have revised our manuscript according to the comments and recommendations of the reviewers. We have highlighted all changes in the revised manuscript in **red font**, and a clean copy of the manuscript is also uploaded. Below, we have included an itemized series of responses to the comments of the reviewers.

Reviewer #1:

**1. Title: “Patient Acuity Rating by Nurses in Rapid Response Team”. The title is inappropriate; I think it would be better to address what you intend to do in the study which is score evaluation in the population you made because with this title it looks like a review article e.g evaluation, assessment**

Response) We appreciate your comment. After careful discussion between the authors, we have decided to change to title of the article as: “Performance of Patient Acuity Rating by Rapid Response Team Nurses for Predicting Short Term Patient Prognoses.”

**2. Abstract: Not acceptable in the current format, the abstract is poorly written, there is no background. I suggest the following to improve the abstract. The background should not describe the score the authors intend to study, but it should include literatures to support the hypothesis. Aim of the work is not clear**

Response) We agree with your comment. We have deleted unnecessary descriptions, and have clarified the aim of our study.

***“Background:*** *Although scoring and machine learning methods have been developed to predict patient deterioration, bedside assessment by nurses should not be overlooked. This study aimed to evaluate the performance of subjective bedside assessment of the patient by the rapid response team (RRT) nurses in predicting short-term patient deterioration.”*

**3. Methods : the authors should not include the statement “is that this work is based on a retrospective review of a prospective observational cohort” This make the reader loss interest indeed they should include methodological summary, a short description of the acuity score, if they used additional instruments, I think the study intend to test two scores which should come here, sample volume type of the study should come in the methodology, the study subjects, should include the type of sampling.**

Response) Thank you for your comment. We have deleted the statement “this work is based on a retrospective review of a prospective observational cohort,” and added a short description of the study subjects, along with patient acuity score and other early warning scores.

***“Methods:*** *Patients noticed by RRT nurses based on the vital sign instability, abnormal laboratory results, and direct contact via phone between November 1, 2016, and December 12, 2017, were included. Five RRT nurses visited the patients according to their shifts and assessed the possibility of patient deterioration. Patient acuity rating (PAR), a scale of 1–7, was used as the tool of bedside assessment. Other scores, including the modified early warning score, VitalPAC early warning score, standardised early warning score, and cardiac arrest risk triage, were calculated afterwards. The performance of these scores in predicting mortality and/or intensive care unit admission within 1 day was compared by calculating the area under the receiver operating curve.”*

**4. The population of the study is not described whether they are nurse or nurses and physicians, I think this critical, the authors focused on the patient’s outcome however there is a factor related to the observer, so the observer data should be described and included in the statistical analysis**

Response) The observers in our study are nurses from the RRT, and we have clarified our sentences.

***“Methods:*** *… Five RRT nurses visited the patients according to their shifts and assessed the possibility of patient deterioration. Patient acuity rating (PAR), a scale of 1–7, was used as the tool of bedside assessment. …”*

**5. Results: the results should start with descriptive numbers from the study population followed by the rest, the authors did not use abbreviation for the score also it is mentioned many times, I do not know why they use capital letters in multiple occasions.**

Response) The numbers from the study population, along with those with main outcomes (composite outcome, defined as death or admission to the ICU within the next day of RRT activation) were added. The scores were abbreviated as they were mentioned in multiple occasions.

***“Results:*** *A total of 1,426 patients were included in the study, of which 258 (18.1%) died or were admitted to the intensive care unit within 1 day. The area under the receiver operating curve of PAR was 0.87 (95% confidence interval [CI] 0.84–0.89), which was higher than those of modified early warning score (0.66, 95% CI 0.62–0.70), VitalPAC early warning score (0.69, 95% CI 0.66–0.73), standardised early warning score (0.67, 95% CI 0.63–0.70) and cardiac arrest risk triage (0.63, 95% CI 0.59–0.66) (P<.001). The area under the receiver operating curve of PAR tended to be larger for nurses with longer experience in the RRT.”*

**6. Conclusions: The conclusion should summarize your results in a better attractive way**

Response) We agree that unnecessary sentences were included in the conclusion. It was summarized as following:

***“Conclusions:*** *PAR assessed by RRT nurses can be a useful tool for assessing short-term patient prognosis in the RRT setting.”*

**7. I think that abstract needs to be revised by a professional English editor**

Response) We have revised our manuscript with help of a professional English editor website (https://www.editage.co.kr/).

**8. The introduction suffers a lot of redundancy, it should be more focused. The introduction should include the background of the importance of decisions by the rapid response team. The score does not needs to be described in this section but its importance in different population should be highlighted**

Response) We agree with you about the redundant expressions in the manuscript. Unnecessary sentences were deleted, and we have emphasized the importance of early detection and management of critically ill patients by the RRT. Detailed description about patient acuity rating was moved to the methods section.

***“Introduction***

*Rapid response team (RRT) is a multidisciplinary team staffed by healthcare professionals with expertise in critical care. Although RRTs provide variable reductions in patient mortality rates, delayed response remains one of the strongest predictors of mortality and unexpected intensive care unit (ICU) admission among critically ill patients [1-3]. Early detection and decisions made by the RRT to correctly triage the patient are essential. The RRT must communicate with medical staff in the general ward, determine whether further medical back-ups are required, and manage the patient during this time.*

*To facilitate early detection of deteriorating patient status, various early warning scores have been developed. For example, the modified early warning Score (MEWS), one of the most well-known early warning scores, is used in several clinics to facilitate decision-making [2, 4, 5]. However, MEWS provides variable accuracy, and it remains unclear whether the score can be used alone to predict unexpected critical events [3, 6]. Other scoring systems, including VitalPAC early warning score (ViEWS) [7], standardised early warning score (SEWS) [8], and the cardiac arrest risk triage (CART), have also been introduced; however, they were revealed to have performance similar to that of MEWS [3]. These factors have led to the development of several machine learning methods and new risk stratification tools, although the complexity of these techniques can make it difficult to implement these in a pragmatic manner [9, 10].*

*Patient acuity rating (PAR), a 7-point scale, has been introduced as a method of subjective bedside assessment of the patient [11]…”*

**9. The authors highlighted that they intend to “This study aimed to evaluate the accuracy of the PAR compared to other early warning scores when scored by RRT” the other scoring system needs to be mentioned like A versus B**

Response) We understand your concern. We aimed to compare multiple scores against patient acuity rating: modified early warning score, VitalPAC early warning score, standardised early warning score, and cardiac arrest risk triage. We have amended the sentence as following:

*“This study aimed to evaluate the performance of the PAR when scored by RRT nurses, versus other early warning scores”*

**10. The methodology as the rest of the manuscript suffers a critical lack of focusing issues. The authors do not need to mention that this study is based on the others work, they should cite to this score in the introduction and start the methodology by what they did, the work is considered original if it is conducted in another population and or different sample volume.**

Response) We appreciate your careful reading, but our study was not based on others’ work: it was from our center. We have deleted the statement “this work is based on a retrospective review of a prospective observational cohort,” and amended the sentences to minimize the misunderstanding by the readers.

***“Materials and Methods***

*Study design and patients*

*This study included adult patients who required RRT support during their admission between November 1, 2016, and December 12, 2017.”*

**11. a figure including the score they used is needed for the reader “available at” J Hosp Med. 2011 October; 6(8): 475–479. doi:10.1002/jhm.886.**

Response) We agree with your comment. However, due to the copyright issues, the figure itself could not be used in our manuscript. Therefore, we added a sentence in the methods section as following:

*“... PAR of 1 corresponds to the lowest probability of patient deterioration and 7 corresponds to the highest. A diagram for better understanding of PAR is available from the original study [11].…”*

**12. The population of the study is not clearly described whether they are nurse or nurses and physicians, I think this critical, the authors focused on the patient’s outcome however there is a factor related to the observer diversity, so the observer data should be described and included in the statistical analysis. The number also is essential, the demographics within the group is important e.g within the analyzing nurses what is the total years of experience, whether they share the same precision of assessment**

Response) We apologize for the ambiguous description. Nurses were the observers who checked patient acuity rating among critically ill patients contacted to the RRT. Physicians did not participate in the scoring process. We have changed the subtitle in the methods section from “Composition of the RRT” to “Nurses in the RRT.” Detailed information about each nurse are described as following in the manuscript:

*“Nurses in the RRT*

*Five nurses, who were co-authors of our study, were included in the RRT. All nurses had >9 years of experience of working as a nurse and at least 5 years of experience in the ICU. Nurses 1, 2, and 3 had a >4-year experience of working in the RRT whereas nurses 4 and 5 had a <2-year experience of working in the RRT. Nurses 1, 2, 3, and 4 had completed an ICU nursing program, a nurse preceptor program, and a basic life support provider program. Nurses 1 and 2 had completed an advanced cardiac life support provider program, and nurse 2 was working as an instructor for a basic life support provider program. The RRT nurses did not have direct authority on the final medical decision.”*

**13. reliability and validity score for the used questionnaires had not been identified this needs to be checked by a statistician, which include a secondary analysis related to the observers**

Response) Thank you for your comment. The article was checked by a qualified statistician from Medical Research Collaborating Center of Seoul National University Bundang Hospital.

To provide the reliability of PAR in predicting the composite outcome, we drew a calibration plot with Hosmer-Lemeshow test for goodness-of-fit. Calibration plot seemed feasible, but Hosmer-Lemeshow test revealed *p*-value of 0.039 with group of 7, and 0.253 with group of 6. The calibration plot was included as Fig 2, and the main text was revised as following:

*“Methods*

*Study outcomes and statistical analysis*

*…The AUROCs for PAR and other scoring systems were compared using the DeLong method with Bonferroni-adjustment.* *Because PAR involves subjective assessment of patient status, the AUROCs of PAR for predicting the composite outcome according to each participating nurse were analyzed. A combined model of PAR with ViEWS, which showed the largest AUROC among the calculated scores, was built with logistic regression analysis, and the AUROC of this model was calculated as well. To assess the goodness-of-fit of a regression model, calibration plot was drawn with Hosmer-Lemeshow test.*

…

*The statistical methods were checked by a qualified statistician from Medical Research Collaborating Center of Seoul National University Bundang Hospital.*

*Results*

*Comparison of PAR against other scores*

*… A cut-off value of PAR ≥4 results in sensitivity of 84.9% and specificity of 73.2% for predicting the composite outcome. The sensitivity and specificity of PAR for various cut-off points are described in Table 3. Calibration plot of PAR for predicting the probability of the composite outcome seemed feasible (Fig 2), but Hosmer-Lemeshow test revealed P of 0.039 with group of 7, and 0.253 with group of 6.*

***Fig 2. Calibration plot of Patient Acuity Rating for predicting the probability of the composite outcome”***

Validity (performance) of PAR in predicting the composite outcome was the main outcome our study tried to assess, by comparing the area under the receiver operating characteristic curve versus other scoring systems. PAR was introduced as a subjective method of assessment of patient deterioration from a previous study (Edelson DP et al. J Hosp Med, 2011), therefore our population for validation is different from the originally derived population. The validity of other early warning scores has been introduced as area under the receiver operating characteristic curve in a previous review (Churpek MM et al. Chest, 2013).

**14. The results should be revised with new analysis including the observer’s data as requested**

Response) Thank you for the comment. The results according to each observer (nurse) is described in Table 4, and the results section as following:

*“PAR assessment according to each nurse*

*The AUROCs were larger for nurses 1, 2 and 3 with longer experience in the RRT (AUROC, 0.91 95% CI 0.87–0.95], 0.91 [95% CI 0.85–0.96] and 0.90 [95% CI 0.86–0.94] respectively) than for nurses 4 and 5, with shorter experience (AUROC, 0.78 [95% CI 0.72–0.84] and 0.80 [95% CI 0.71–0.90] respectively). AUROC of PAR for nurse 4 was significantly smaller compared to those of nurses 1, 2 and 3.*

*To compensate for the smaller AUROC of less-experienced nurses’ PAR, we utilized a logistic regression model of PAR with the warning score of the largest AUROC in our study: ViEWS. The combined model of PAR and ViEWS in the overall population showed a significantly improved AUROC (0.875 [95% CI, 0.849–0.900]) compared to that of PAR alone (0.868 [95% CI 0.843–0.894]). This change was mainly due to the improvement in AUROC for nurse 4; the combined model showed a significantly improved AUROC (0.81 [95% CI 0.75–0.86]) for predicting patient outcome compared to that of PAR alone (0.78 [95% CI 0.72–0.84]). Meanwhile, significant improvement of AUROC in this model was not observed for the other four nurses (Table 4).”*

**15. Statistical review: the manuscript needs to be seen by a statistician. The study outcome section needs to incorporated into the statistical analysis**

Response) We appreciate your comment. The article was checked by a qualified statistician from Medical Research Collaborating Center of Seoul National University Bundang Hospital. Fig 2 was added to further assess the reliability of Patient Acuity Rating in predicting the composite outcome, and the term “performance” was used rather than “accuracy.” DeLong method with Bonferroni-adjustment for comparing the area under the receiver operating characteristic curve was double checked by the statistician. A sentence was added to the methods section as following:

*“The statistical methods were checked by a qualified statistician from Medical Research Collaborating Center of Seoul National University Bundang Hospital.”*

**16. I think the manuscript needs major revision by the authors to decide upon how the discussion will go. The discussion is too long and it should start with the salient findings followed by focused analysis. The discussion needs 2nd round of revision after fixing the earlier issues**

Response) We apologize for the redundant expressions. The discussion section was shortened, only leaving necessary sentences.

**17. Quality of written English: Need secondary revision**

Response) We have revised our manuscript with help of a professional English editor website (https://www.editage.co.kr/).

**18. Ethical concerns: The author explained that they obtained waived ethical approval from their institute regarding the patients but they did should include approval related to the participation of nurses**

Response) We appreciate your comment. The five nurses are co-authors of our manuscript (Hyun-Ju Min, Dong-Seon Lee, Yun-Young Choi, Miae Yoon, and Da-Yun Lee), who all agreed to the design of the study and the final version of the manuscript. It was added to the methods section as following:

*“Nurses in the RRT*

*Five nurses, who were co-authors of our study, were included in the RRT.”*

**19. The references style was not adequately followed according to Plos One style.**

Response) We have double checked our references to be concordant with Plos One style.

**Reviewer #2:**

**MAJOR COMMENTS**

**1. More information is needed on the methods e.g. inclusion/exclusion criteria for both patients and nurses and on what basis the sample size was determined?**

Response) We appreciate your comment. The inclusion/exclusion criteria, along with sample size calculation was included in the manuscript. Sample size was calculated by estimating AUROC of PAR as 0.78 according to a pilot analysis of patients from January 2015 to March 2016 in our center, and AUROC of MEWS as 0.72 according to a previous report (Subbe CP et al. Qjm. 2001;94(10):521-6). With alpha and beta set as 0.05 and 0.20 respectively, the calculated sample size was 1420.

***“Materials and Methods***

*Study design and patients*

*This study included adult patients who required RRT support during their admission between November 1, 2016, and December 12, 2017. …*

*Sample size was calculated by estimating the area under the receiver operating characteristic curve (AUROC) of PAR as 0.78, according to a pilot analysis of patients from January 2015 to March 2016 in our center, and the AUROC of MEWS as 0.72, according to a previous report [4]. The RRT was activated by the following criteria: systolic blood pressure <90 mmHg; heart rate <50/min or >140/min; respiratory rate <10/min or >30/min; body temperature >39 °C or <36 °C; peripheral oxygen saturation <90% with room air and/or facial mask with oxygen flow >8 L/min; serum pH <7.3; serum partial pressure of carbon dioxide >50 mmHg; serum partial pressure of oxygen <60 mmHg; serum lactic acid >2.5 mmol/L; serum total carbon dioxide <15 mmol/L; or direct concerns from ward nurses. Patients with sudden cardiac arrest and those in whom PAR was not assessed were excluded from the study. In patients with multiple instances of RRT support, only the first instance was included in the analysis.”*

**2. Were all the scores independent cases i.e. individual patients?**

Response) We apologize for the ambiguous description. All the scores were individual patients. If one patient was notified to the RRT multiple times, only the first case was included in the study. The clarified sentence was added to the methods section:

*“… Patients with sudden cardiac arrest and those in whom PAR was not assessed were excluded from the study. In patients with multiple instances of RRT support, only the first instance was included in the analysis. …”*

**3. Line 72: It’s not clear what a “retrospective review of a prospective cohort study” means. What were the reasons for the delay and what was the duration of this between the prospective data collection and the retrospective review? Please clarify and where possible provide a reference for the prospective cohort study on which the retrospective review was based.**

Response) Thank you for your comment. This study was not based on others’ work: it was from our center. We have deleted the statement “this work is based on a retrospective review of a prospective observational cohort,” and amended the sentences to minimize the misunderstanding by the readers.

***“Materials and Methods***

*Study design and patients*

*This study included adult patients who required RRT support during their admission between November 1, 2016, and December 12, 2017. …*

*Sample size was calculated by estimating the area under the receiver operating characteristic curve (AUROC) of PAR as 0.78, according to a pilot analysis of patients from January 2015 to March 2016 in our center, and the AUROC of MEWS as 0.72, according to a previous report [4]. The RRT was activated by the following criteria: systolic blood pressure <90 mmHg; heart rate <50/min or >140/min; respiratory rate <10/min or >30/min; body temperature >39 °C or <36 °C; peripheral oxygen saturation <90% with room air and/or facial mask with oxygen flow >8 L/min; serum pH <7.3; serum partial pressure of carbon dioxide >50 mmHg; serum partial pressure of oxygen <60 mmHg; serum lactic acid >2.5 mmol/L; serum total carbon dioxide <15 mmol/L; or direct concerns from ward nurses.* *Patients with sudden cardiac arrest and those in whom PAR was not assessed were excluded from the study. In patients with multiple instances of RRT support, only the first instance was included in the analysis.”*

**4. Lines 98-101 – were the PAR scores and the four early warning sign scores shared with the intensivists who made the decisions about ICU admission on those occasions when the nurse first raised the alarm?**

Response) We understand your concern. All of the scores mentioned in our study were not known to the intensivists who made the decisions about ICU admission. It was mentioned in the methods section as following:

*“… The RRT nurses and physicians were not aware of these scores at the time of their visits and assessments, and the scores did not influence clinical decisions ….”*

**5. Line 124: please give more detail on the logistic regression methods i.e. how variables were selected, entered, and removed from, the final models.**

Response) VitalPAC early warning score was selected to be combined with patient acuity rating, because it showed the largest area under the receiver operating characteristic curve compared to other warning scores (modified early warning score, standardised early warning score, and cardiac arrest risk triage). We agree that the previous description may mislead the readers, therefore we have revised the sentences as following:

*“… Because PAR involves subjective assessment of patient status, the AUROCs of PAR for predicting the composite outcome according to each participating nurse were analyzed. A combined model of PAR with ViEWS, which showed the largest AUROC among the calculated scores, was built with logistic regression analysis, and the AUROC of this model was calculated as well. …”*

**6. Line 169: which statistical test was used to generate this probability level? None of the statistical tests described in the Methods seem appropriate. ANOVA or Kruskal-Wallis would be the tests usually employed for multiple group comparisons such as this.**

Response) The areas under the receiver operating characteristic curve were compared using the DeLong method, with Bonferroni-adjustment. The information was added to the methods section as following:

*“… The AUROCs for PAR and other scoring systems were compared using the DeLong method with Bonferroni-adjustment. …”*

**7. Adding some discussion of the similarities and differences between the authors’ PAR results and those of related studies (e.g. Edelson et al (2011) [ref 10] and O’Donnell et al (2016) [ref 11]) is important in order to place the findings in the context of the available literature - similar to the way the authors discuss their early warning score findings commencing at line 268.**

Response) We appreciate your great idea. The study by Edelson et al (2011) was mentioned, which revealed the area under the receiver operating characteristic curve of patient acuity rating of 0.82, which was scored by physicians.

*“… Our results highlight that non-physician healthcare professionals are helpful in the RRT setting. A previous study has demonstrated the AUROC of PAR assessed by physicians to be 0.82 (0.69 for residents and 0.85 for attendings) for predicting short-term patient deterioration [11], which is comparable to the AUROC in this study (0.87 [95% CI 0.84–0.89]). …”*

**8. The authors should acknowledge the limitations of their data when discussing the importance of nurse work experience and the accuracy of PAR scores. Specifically, they identified two nurses with less RRT experience whose PAR score accuracy was compared with three more experienced RRT nurses. Such a small sample size (of nurses) cannot provide an adequate test of the impact of experience on PAR accuracy so greater caution in generalizing this finding is called for. The least experienced nurses might have had other characteristics which were of equal or greater relevance to the question of their PAR accuracy.**

Response) We deeply agree with you on the issue. The small sample size of nurses (5 nurses) makes statistical analysis not viable. Although our study findings cannot be generalized to other nurses with diverse backgrounds, it may suggest that sufficient working experience as a RRT nurse is an important component of a nurse’s ability to correctly predict patient prognosis. A sentence in the discussion section was amended as following:

*“…* *One of the major drawbacks of PAR is its subjective nature. As shown in our study, the reliability of PAR was questionable: PAR of nurses with longer experience in the RRT revealed an AUROC of over 0.9 in predicting mortality and/or ICU admission within the next day, but those with less experience (<6 months to 1 year) showed an AUROC of under 0.8. Although the small number of nurses makes statistical comparison impossible, it seems that sufficient work experience as an RRT nurse may be an important component of a nurse’s ability to correctly predict patient prognosis. …”*

**9. Lines 281-283: the authors rightly identify a crucial limitation of the design i.e. that the nurses involved were not independent assessors of patient acuity but involved in the triage process itself, which would likely inflate the AUROCs of the PAR scores in relation to the admission to ICU outcome. It would be helpful if the authors could reflect at this juncture on improved aspects of design which would provide a more robust test of nurse-scored PAR accuracy in this clinical context.**

Response) Thank you for your careful reading. Future studies with blinded researchers may further assess the accuracy of PAR. A sentence was added to the discussion section mentioning the limitations as following:

*“… Second, although final decision of patient management was made by the notified physician, the RRT nurse’s subjective opinion could have influenced the physician’s decisions, which might have led to more frequent ICU admissions for patients with high PAR. To exclude such potential influences, PAR needs to be assessed by nurses outside the RRT pathway in future studies. …”*

**MINOR COMMENTS**

**10. Lines 52-59: A slightly more comprehensive summary of the early warning score methods available would strengthen the Introduction – especially so if addressing the pros and cons of all four of the scores used in the study.**

Response) We agree with your comment. Mentioning of other early warning scores was added to the introduction as following:

*“To facilitate early detection of deteriorating patient status, various early warning scores have been developed. For example, the modified early warning Score (MEWS), one of the most well-known early warning scores, is used in several clinics to facilitate decision-making [2, 4, 5]. However, MEWS provides variable accuracy, and it remains unclear whether the score can be used alone to predict unexpected critical events [3, 6]. Other scoring systems, including VitalPAC early warning score (ViEWS) [7], standardised early warning score (SEWS) [8], and the cardiac arrest risk triage (CART), have also been introduced; however, they were revealed to have performance similar to that of MEWS [3]. These factors have led to the development of several machine learning methods and new risk stratification tools, although the complexity of these techniques can make it difficult to implement these in a pragmatic manner [9, 10].”*

**11: The authors chose to compare the AUROC of the PAR, a subjective measure, with four objective measures derived from clinical signs. Why did they choose these particular objective measures (see Comment 10)? Did they explore the possibility of making comparisons between PAR and other subjective measures of patient acuity? Explanation of the rationale in both cases would be informative in assessing the validity of the design.**

Response) Thank you for your careful reading. The main aim of this study was to assess the performance of subjective assessment of the patients by RRT nurses. In order to do so, we decided to compare patient acuity rating against other well-known early warning scores, including modified early warning score, VitalPAC early warning score, standardised early warning score, and cardiac arrest risk triage. Comparing patient acuity rating against other subjective measures was not appropriate, because the main aim of our study was to assess the performance of the subjective measure, which has not been studied before.

*“During the study period, the RRT nurses evaluated the probability of patient deterioration using the PAR, from a scale of 1 to 7 [11].* *PAR of 1 corresponds to the lowest probability of patient deterioration and 7 corresponds to the highest. A diagram for better understanding of PAR is available from the original study [11]. Other well-known scores for predicting short-term patient deterioration, including MEWS, ViEWS, SEWS, and the CART score, were calculated afterwards.”*

**12. Line 131: “Patterns of distributions were assessed by visual inspection” – what was the rationale for not testing the normality and heterogeneity of variance of the distributions using commonly used tests?**

Response) We appreciate your comment. After Shapiro-Wilk test, “Age” and “Body mass index” revealed not to follow the normal distribution. Therefore, the descriptions were changed into median and interquartile ranges throughout the manuscript.

**13. Line 139: “composite outcome” – please clarify the meaning of this phrase at this point.**

Response) Thank you for your concern. The sentence was amended as following:

*“During the study period, 1,441 patients triggered the RRT. Nine patients with sudden cardiac arrest and six patients whom PAR was not assessed were excluded. Therefore, 1,426 patients were included in the final analysis. Among them, 258 patients (18.1%) experienced death and/or ICU admission within 1 day, defined as the “composite outcome”.”*

**14. Line 146: “when the RRT was contacted directly” – please explain what this means – what were the other means by which the RRTs were engaged?**

Response) We apologize for the ambiguous description. It meant that they were contacted directly by phone because of concerns other than specified abnormalities of vital signs and laboratory findings. The sentence was amended as following:

*“Composite outcome was expected when patients had abnormalities in serum pH (3.9% vs. 0.6%, P<0.001) or when the RRT was contacted directly by phone due to other concerns (18.2% vs. 4.5%, P<0.001).”*

**15. Line 169: Please provide the probabilities associated with the analyses of differences in AUROC’s between the five measures for the ICU admission and mortality outcomes.**

Response) As recommended, we added the probabilities associated with the analyses into the main manuscript.

*“PAR was superior in anticipating ICU admission (AUROC, 0.87 [95% CI, 0.84–0.89]) than MEWS (AUROC, 0.65 [95% CI, 0.61–0.69]), ViEWS (AUROC, 0.68 [95% CI 0.64–0.72]), SEWS (AUROC, 0.66 [95% CI 0.62–0.70]), and CART (AUROC, 0.63 [95% CI 0.59–0.67]) but was not superior in predicting mortality (AUROC, 0.79 [95% CI 0.70–0.87]) than MEWS (AUROC, 0.69 [95% CI 0.61–0.76]), ViEWS (AUROC, 0.70 [95% CI 0.61–0.78]), SEWS (AUROC, 0.69 [95% CI 0.62–0.77]), and CART (AUROC, 0.58 [95% CI 0.49–0.67]) (Table 2).”*

**16. Lines 193-196 and Lines 200-202: Please quote the probabilities associated with these three analyses.**

Response) As recommended, we added the probabilities associated with the analyses into the main manuscript.

*“The combined model of PAR and ViEWS in the overall population showed a significantly improved AUROC (0.875 [95% CI, 0.849–0.900]) compared to that of PAR alone (0.868 [95% CI 0.843–0.894]). This change was mainly due to the improvement in AUROC for nurse 4; the combined model showed a significantly improved AUROC (0.81 [95% CI 0.75–0.86]) for predicting patient outcome compared to that of PAR alone (0.78 [95% CI 0.72–0.84]).”*

**17. The analyses by individual nurses, taking into consideration their levels of experience, and the logistic regression modelling of the predictive value of PAR and ViEWS in combination bring added value to the design and are of practical significance.**

Response) We appreciate your comment.

**18. Are the sentences at lines 215 and 223 not contradictory?**

Response) Sentence at line 215 tried to emphasize that this was the first study to be performed in the “RRT setting.” Sentence at line 223 tried to mention other studies performed in other clinical settings. To reduce the possibility of misleading the readers, we have amended the sentences as following:

*“This is the first study to demonstrate the superiority of PAR over other early warning scores in predicting short-term patient prognosis in the RRT setting, highlighting the importance of bedside patient evaluation implemented in previous studies with different clinical settings [16, 17].”*

**Reviewer #3:**

**Thank you for allowing me to review this study by Kim et al. Rapid response teams are a major development in inpatient care and understanding better ways to objectively triage and assess patients is an important issue. I also think the more we can understand how all providers of care (nurses, physical therapists, physicians) interact with patients is extremely useful. Thus, I read this study with great interest.**

**The authors used a retrospective review of a prospective observational cohort of patients at a single health center and how patients were assessed by rapid response team nurses. They reported finding that a subjective, 7-point Likert scale, the PAR performed better overall than other, more objective scores. The authors also spent a lot of time dissecting the experience of one less-experienced team member versus the other four.**

**I think that there is some interest in the overall finding - PAR performed better than other scores. I am very guarded in my review of the study however because the authors used almost half of the manuscript to discuss essentially a descriptive difference between 2 nurses newer to the team to 3 others who had been on the team for a longer period of time. This study was not designed to assess the experience of the nurses on a rapid response team (statistics cannot be performed this low of a sample size). I thus think this study presentation needs to be rebuilt around the actual findings of the study.**

**Major issues:**

**1. There is no power to assess for the experience of nurses and the authors report experience of nurses as a major conclusion of the entire study**

**There are 3 nurses with >4 years’ experience as an RRT nurse. There are 2 with 1 year or less experience. Only 1 of 2 nurses with less experience (Nurse 4) had a significantly lower AUROC than the 3 more experienced nurses (Nurses 1-3). There is no way to make even a subjective conclusion off a single individual underperforming (Nurse 4) and the other individual performing the same (Nurse 5). I don’t even think it belongs in the manuscript beyond perhaps a description of the 5 nurses in the RRT team. I would draw the conclusion the nurses performed the same except for one. They also had less training, but another nurse with even less training (Nurse 5) performed the same.**

**Because this is reported as a major conclusion of the study throughout, including the abstract, this entire manuscript needs to be refocused on the actual objective study findings before it can be fully vetted.**

Response) We deeply understand your concern. Although the experience of the assessing RRT nurse may be an import factor for higher performance of PAR, we agree that small number of nurses in this study makes it difficult to include the findings as a major conclusion. After careful discussion between the authors, we have decided to delete the sentences about experience of nurses as a major conclusion from both the abstract and the main text. The text was amended as following:

***“Conclusions:*** *PAR assessed by RRT nurses can be a useful tool for assessing short-term patient prognosis in the RRT setting.*

*…*

***Conclusions***

*In conclusion, subjective assessment of the patient by the RRT nurse, represented as PAR reveals good performance in predicting patient prognosis. Although early warning scores may be useful for identifying at-risk patients, direct examinations by healthcare professionals should be emphasized when RRT is activated.”*

**2. I am not sure the comparison scores are the best available**

**The authors admit that MEWS was not developed for general ward patients (it was developed for acute medical admissions). The scores also looked at different outcomes. Why did the authors choose these scores? Is it fair to say the PAR score outperforms scores that are not clearly validated in the actual patient population? Is this important? ViEWS and CART seem fair, but SEWS, and most certainly MEWS, seem less applicable. Are there better scores to compare? The authors calculate the Charlton Comorbidity Index as important but did not compare the PAR with this score. I think it would be helpful.**

Response) We appreciate your comment. Although the scores were created from diverse clinical settings, the scores used in our study (MEWS, ViEWS, SEWS, and CART) were meant to anticipate short-term patient prognosis. Furthermore, the area under the receiver operating characteristic curve (AUROC) of patient acuity rating (PAR) was calculated to be over 0.8 in our study, which refers to good power of prediction by itself.

Charlson Comorbidity Index (CCI), on the other hand, is meant to predict 10-year mortality. Therefore, comparing CCI against PAR does not seem to be appropriate. The area under the receiver operating characteristic curve of CCI is calculated to be 0.485 in our population, to predict mortality and/or ICU admission within the next day.

Sentences were added to the manuscript as following in the discussion section:

*“Our study identified smaller AUROC values for other early waring scores compared to previous studies [3, 4, 7, 14, 15]. This is likely related to the studies being performed in different medical settings. MEWS was validated in the acute medical admissions setting, and nurses collected the scoring variables during their routine duties [4]. ViEWS and SEWS were studied in the emergency treatment setting [7, 8], and CART was validated in the general ward [15]. Moreover, these tools targeted different clinical outcomes in each studies: admission to a higher dependency unit, attendance of the cardiac arrest team, death and survival at 60 days (MEWS) [4]; in-hospital mortality within 24 hours (ViEWS) [7]; in-hospital mortality and length of stay (SEWS) [8]; and in-hospital cardiac arrest and ICU admission (CART) [15]. These differences could create a noticeable difference in the AUROC values produced, as our study calculated AUROC with selected patients who activated the RRT, in which worse prognosis is anticipated compared to the normal patient population. However, this does not devaluate the power of PAR in our study; the AUROC of PAR was 0.87 (95% CI 0.84–0.89), which refers to good power for predicting short-term patient deterioration.”*

**3. What was the best cut-off to predict ICU admission for the PAR in this study?**

**I was a bit confused that the PAR score is deemed more reliable a determination for predicting bad outcomes in patients who have an RRT consultation, however, it was not expressly clear to me what cut-off was used to predict bad outcome most accurately. Certainly, if one is to apply the use of PAR for clinical practice, there should be some cutoff point for providers to follow as to should the patient be admitted to the ICU. The authors need to discuss this as it is critical for actual applying a diagnostic test.**

Response) We appreciate your careful reading. A cut-off value of 4 may be a reasonable point with good sensitivity (84.9%) and fair specificity (73.2%) for predicting short-term patient deterioration. It was added to the discussion section as following:

*“Our results highlight that non-physician healthcare professionals are helpful in the RRT setting. A previous study has demonstrated the AUROC of PAR assessed by physicians to be 0.82 (0.69 for residents and 0.85 for attendings) for predicting short-term patient deterioration [11], which is comparable to the AUROC in this study (0.87 [95% CI 0.84–0.89]).* *Recent reductions in resident duty hours (80-hour maximum weekly limit) by the Accreditation Council for Graduate Medical Education have created longer handoff periods between inpatient physicians [20], which leads to an increased need for non-physician healthcare professionals. In combination with early warning scores, the use of subjective patient assessments by ward nurses can provide a better ability to predict patient deterioration [21]. Considering the sensitivity and specificity of PAR (Table 3), a cut-off value of ≥4 can be reasonable, with good sensitivity and fair specificity for predicting short-term patient deterioration.”*

**4. One combination assessment was tested – PAR + ViEWS – but not others**

**It was reported in Table 4 about the individual nurse subjects’ performance on PAR, ViEWS and PAR+ViEWS. As stated above, the comparison between nurses for experience is a minor point. It would be much more useful to assess if combining the PAR with the ViEWS in the overall cohort was superior to either alone.**

Response) We appreciate your comment. We compared PAR versus PAR+ViEWS in the overall population. PAR+ViEWS reveal larger area under the receiver operating characteristic curve compared to PAR alone, but this is mainly due to “nurse 4.” The difference was only significant in patients assessed by “nurse 4,” but not in patients assessed by other nurses. Therefore, the superiority of PAR+ViEWS was due to “nurse 4,” rather than other nurses. The findings were added to the results section as following:

*“To compensate for the smaller AUROC of less-experienced nurses’ PAR, we utilized a logistic regression model of PAR with the warning score of the largest AUROC in our study: ViEWS.* *The combined model of PAR and ViEWS in the overall population showed a significantly improved AUROC (0.875 [95% CI, 0.849–0.900]) compared to that of PAR alone (0.868 [95% CI 0.843–0.894]). This change was mainly due to the improvement in AUROC for nurse 4; the combined model showed a significantly improved AUROC (0.81 [95% CI 0.75–0.86]) for predicting patient outcome compared to that of PAR alone (0.78 [95% CI 0.72–0.84]). Meanwhile, significant improvement of AUROC in this model was not observed for the other four nurses (Table 4).”*

**5. Authors draw conclusions about nurses in general, but only tested five RRT nurses and did not compare performance to other providers in the same cohort of patients**

**The authors draw many conclusions on nurses’ overall assessments versus the performance of other providers (physicians, for example), however this is a highly trained and experienced subset of nurses. There was also no comparison between different types of providers (advanced practice providers, interns, residents, attending physicians) in this particular cohort of patients. To truly make a comparison of these types of providers ability to assess the PAR accurately, their performance on the PAR would need to examined in the same cohort of patients. I think minimal conclusions in direct comparison can be drawn from this study. I also think generalizing the performance of RRT nurses to all nurses throughout the hospital, with a wide subset of skills and specialties, is impossible.**

Response) We admit the limitation of our study, and agree that our findings may not be generalized to other less-experienced nurses. With help of a qualified statistician, the reliability of PAR in predicting the composite outcome was assessed with calibration plot and Hosmer-Lemeshow test, which revealed poor reliability. Patient acuity rating was assessed only by RRT nurses in our study, therefore, comparison between other providers was not possible. We have added several sentences to the limitation, and have made careful modifications to the conclusion as following:

*“This study has several limitations. First, this study was a single-center design which includes only 5 RRT nurses with long working experience, and our results may not be generalized to other less experienced nurses. Inter-observer variability should be considered, and further studies on healthcare professionals with various backgrounds can enforce the strength of PAR.* *Second, although final decision of patient management was made by the notified physician, the RRT nurse’s subjective opinion could have influenced the physician’s decisions, which might have led to more frequent ICU admissions for patients with high PAR. To exclude such potential influences, PAR needs to be assessed by nurses outside the RRT pathway in future studies. Third, this study included patients who have certain extent of systemic illness. This may influence the AUROC of PAR and early warning scores.*

*…*

*In conclusion, subjective assessment of the patient by the RRT nurse, represented as PAR reveals good performance in predicting patient prognosis. Although early warning scores may be useful for identifying at-risk patients, direct examinations by healthcare professionals should be emphasized when RRT is activated.”*

**6. Were any further outcomes assessed? If ICU admission at a later time is an outcome, what was the overall outcome of the patients?**

**Not all ICU admissions are appropriate and more evidence is coming out that perhaps needless ICU admissions are without harm. I also am interested that mortality was actually not significantly predicted by the PAR, which might suggest the ICU admissions did not correlate with mortality as much. Was there any more data to be gained? I would be very interested in overall in-patient mortality. Additionally, if available, 6 month and 1-year mortality would be very useful, although I realize this may be difficult to obtain.**

Response) We appreciate your idea. However, the data of 6-month and 1-year mortality could not be obtained. The main aim of our study was to anticipate short-term patient prognosis.

**Minor issues:**

**1. In table 1, need to put sex, female statistics (how many total, death/ICU admission, alive without ICU admission within 1 day)**

Response) We have added female statistics into Table 1.

**2. What was the p value comparing in sex, age, BMI Charlson comorbidity index in Table 1? Was it if that variable predicted outcome on logistic regression? State this in the table details.**

Response) The *p*-values were added to the manuscript. After logistic regression analysis, BMI was associated with the composite outcome (odds ratio 0.97, 95% confidence interval 0.94–1.00, per kg/m2 of BMI, *P*-value = 0.044). However, BMI was not known to the RRT nurse at the time of PAR assessment, therefore did not influence PAR.

*“The patients exhibited male predominance (60.6%), a median age of 72 (IQR, 61–79) years, a mean body mass index of 21.4 (IQR, 19.4–24.7) kg/m^2^, and a median Charlson comorbidity index of 2 (IQR, 1–4). Patients who died and/or admitted to the ICU seemed to have lower body mass index than those who did not (median 21.2 vs. 22.0, P-value=0.020). Sex, age, and Charlson comorbidity index did not differ significantly between the two groups (P-values 0.173, 0.310, and 0.427, respectively).”*

**3. Table 1, don’t need to state detailed criteria for rapid response team in the caption**

Response) We agree with your comment. The caption was deleted.

**4. The details of how the RRT work at the hospital is long and extremely detailed. I’m not sure we need all the shift times.**

Response) Thank you for your comment. We have deleted the redundant sentences, and have amended the sentences in the methods section as following:

*“This study included adult patients who required RRT support during their admission between November 1, 2016, and December 12, 2017. …*

*Sample size was calculated by estimating the area under the receiver operating characteristic curve (AUROC) of PAR as 0.78, according to a pilot analysis of patients from January 2015 to March 2016 in our center, and the AUROC of MEWS as 0.72, according to a previous report [4]. The RRT was activated by the following criteria: systolic blood pressure <90 mmHg; heart rate <50/min or >140/min; respiratory rate <10/min or >30/min; body temperature >39 °C or <36 °C; peripheral oxygen saturation <90% with room air and/or facial mask with oxygen flow >8 L/min; serum pH <7.3; serum partial pressure of carbon dioxide >50 mmHg; serum partial pressure of oxygen <60 mmHg; serum lactic acid >2.5 mmol/L; serum total carbon dioxide <15 mmol/L; or direct concerns from ward nurses. Patients with sudden cardiac arrest and those in whom PAR was not assessed were excluded from the study. In patients with multiple instances of RRT support, only the first instance was included in the analysis.*

*Data of baseline demographics, Charlson comorbidity index, department of admission, causes of RRT notification, characteristics of RRT triggering, result of the RRT intervention, and RRT nurse-assessed PAR for the probability of ICU admission and/or mortality within the next day were collected prospectively.*

*…*

*Patient assessment and score calculation*

*During the study period, the RRT nurses evaluated the probability of patient deterioration using the PAR, from a scale of 1 to 7 [11]. PAR of 1 corresponds to the lowest probability of patient deterioration and 7 corresponds to the highest. A diagram for better understanding of PAR is available from the original study [11]. Other well-known scores for predicting short-term patient deterioration, including MEWS, ViEWS, SEWS, and the CART score, were calculated afterwards.*

*…*

*Nurses in the RRT*

*Five nurses, who were co-authors of our study, were included in the RRT. All nurses had >9 years of experience of working as a nurse and at least 5 years of experience in the ICU. Nurses 1, 2, and 3 had a >4-year experience of working in the RRT whereas nurses 4 and 5 had a <2-year experience of working in the RRT. Nurses 1, 2, 3, and 4 had completed an ICU nursing program, a nurse preceptor program, and a basic life support provider program. Nurses 1 and 2 had completed an advanced cardiac life support provider program, and nurse 2 was working as an instructor for a basic life support provider program. The RRT nurses did not have direct authority on the final medical decision.”*

**5. What is a part-time intensivist? Is this a critical care attending physician? Is it a moon-lighter in another specialty? The term is imprecise and I do not know what it means. Also, there was mention of ICU fellows, but in what subspecialty? Critical care medicine?**

Response) Part-time intensivist refers to physicians who participate in the RRT only part-time. They attend to other clinical work such as outpatient clinic when they are off-shift. ICU fellows refer to clinical fellows participating in the management of ICU patients, and they specialize in pulmonology, nephrology, and emergency medicine. Such descriptions seemed redundant, therefore were deleted from the manuscript.

**6. When were clinical variables for the various scores obtained? At bedside or last vital sign check? Same day labs or admission labs?**

Response) The clinical variables for the scores were obtained from the latest alerted vital signs and/or lab findings upon RRT activation, which was on the same day.

*“Details of each score calculation are available in the Tables S1–S4 of the supporting information. Patients’ vital signs used in score calculation were recorded by ward nurses; these data were immediately sent to the RRT.* *The RRT nurses and physicians were not aware of these scores at the time of their visits and assessments, and the scores did not influence clinical decisions.”*

**7. Imprecise language in discussion paragraph 3 (page 12). “the possibility that non-physician healthcare professionals can be helpful in the RRT setting”. Certainly no one would argue that a non-physician healthcare professional is not helpful in an RRT. I don’t understand what the point of the statement is but it could be construed as hurtful to imply the care team is not helpful.**

Response) We agree about the imprecise description. The sentence was amended as following:

*“Our results highlight that non-physician healthcare professionals are helpful in the RRT setting.”*

**8. Discussion paragraph 3, page 12: ACGME duty hours is not referenced. I thought that the ACGME went back to allowing 28-hour shifts for interns. There needs to be an accurate depiction of what the policy is with up to date citations.**

Response) According to the 2017 ACGME common program requirements, a maximum working limit of 80 hours (exceptions for up to 88 hours if educational work was included) is mandatory. Sentences were amended, and appropriate up to date citation was added.

*“Recent reductions in resident duty hours (80-hour maximum weekly limit) by the Accreditation Council for Graduate Medical Education have created longer handoff periods between inpatient physicians [20], which leads to an increased need for non-physician healthcare professionals.”*
