# Supplementary material for: Performance of patient acuity rating by rapid response team nurses for predicting short-term prognosis
Source: PLoS One. 2019 Nov 14;14(11):e0225229. doi: 10.1371/journal.pone.0225229 (PMC6855430; doi:10.1371/journal.pone.0225229)
Supplement: Attachment — Submitted filename: 1_Response_to_editor_and_reviewer_1008.docx [file pone.0225229.s008.docx]

**Dear editor:**

We would like to thank all the editors and reviewers for providing us with their valuable opinions about our manuscript. We have revised our manuscript according to the comments and recommendations of the reviewers. We have highlighted all changes in the revised manuscript in **red font**, and a clean copy of the manuscript is also uploaded. Below, we have included an itemized series of responses to the comments of the reviewers.

**Reviewer #2:**

**Materials and Methods**

**1. Lines 125-132: The response to my original comment “Please give more detail on the logistic regression methods i.e. how variables were selected, entered, and removed from, the final models” has not been adequately addressed. It remains unclear which variables were considered for inclusion in the logistic regression modelling of the composite outcome, and the rationale behind these considerations.**

Response) We appreciate your comment. The combined logistic regression models with PAR and other early warning scores were considered (i.e. PAR + MEWS, PAR + ViEWS, PAR + SEWS, and PAR + CART). Among these, the early warning score with the highest AUROC, ViEWS, was selected to be combined with PAR. We clarified the sentence as follows (Lines 129–132):

*“**A combined model of PAR with other early warning scores (MEWS, ViEWS, SEWS, and CART) were considered for logistic regression analysis. Among them, ViEWS, which showed the largest AUROC among the calculated scores, was selected, and the AUROC of this model was calculated as well.”*

**2. Lines 174-179: Regarding the response to my original comment to “Please provide the probabilities associated with the analyses of differences in AUROC’s between the five measures for the ICU admission and mortality outcomes” the authors have added the estimates and CI’s of the individual early warning sign methods which is to be welcomed – but they have not added the probability levels for either of these tests of group differences in AUROC as they have done for the composite outcome dependent variable.**

Response) Thank you for sharing the great idea. We have added the *p*-values for each comparison: PAR against other warning scores. The sentences were amended as follows (Lines 171–182):

*“In predicting the composite outcome, PAR provided an AUROC of 0.87 with a 95% confidence interval (CI) of 0.84–0.89, which was significantly higher compared to MEWS (AUROC, 0.66 [95% CI, 0.62–0.70]; P<0.001), ViEWS (AUROC, 0.69 [95% CI, 0.66–0.73]; P<0.001), SEWS (AUROC, 0.67 [95% CI, 0.63–0.70]; P<0.001) and CART (AUROC, 0.63 [95% CI, 0.59–0.66]; P<0.001) (Fig 1).* *PAR was superior in anticipating ICU admission (AUROC, 0.87 [95% CI, 0.84–0.89]) than MEWS (AUROC, 0.65 [95% CI, 0.61–0.69], P<0.001), ViEWS (AUROC, 0.68 [95% CI 0.64–0.72]; P<0.001), SEWS (AUROC, 0.66 [95% CI 0.62–0.70]; P<0.001), and CART (AUROC, 0.63 [95% CI 0.59–0.67], P<0.001). Although MEWS was superior in predicting mortality (AUROC, 0.79 [95% CI 0.70–0.87]) than CART (AUROC, 0.58 [95% CI 0.49–0.67]; P=0.001), it was not superior than MEWS (AUROC, 0.69 [95% CI 0.61–0.76]; P=0.062), ViEWS (AUROC, 0.70 [95% CI 0.61–0.78]; P=0.101), or SEWS (AUROC, 0.69 [95% CI 0.62–0.77]; P=0.073) (Table 2).”*

**3. Lines 208-220: Similarly, regarding the response to my original comment “Please quote the probabilities associated with these three analyses” the authors have provided the estimates and CI’s but not the p values.**

Response) Thank you again. The *p*-value was added as follows (Lines 210–214):

*“**The AUROCs were larger for nurses 1, 2 and 3 with longer experience in the RRT (AUROC, 0.91 95% CI 0.87–0.95], 0.91 [95% CI 0.85–0.96] and 0.90 [95% CI 0.86–0.94] respectively) than for nurses 4 and 5, with shorter experience (AUROC, 0.78 [95% CI 0.72–0.84] and 0.80 [95% CI 0.71–0.90] respectively). AUROC of PAR for nurse 4 was significantly smaller compared to those of nurses 1, 2 and 3 (P=0.003).”*

**4. Sentence commencing at Line 272: Regarding the response to my original comment “The authors should acknowledge the limitations of their data when discussing the importance of nurse work experience and the accuracy of PAR scores. Specifically, they identified two nurses with less RRT experience whose PAR score accuracy was compared with three more experienced RRT nurses. Such a small sample size (of nurses) cannot provide an adequate test of the impact of experience on PAR accuracy so greater caution in generalising this finding is called for. The least experienced nurses might have had other characteristics which were of equal or greater relevance to the question of their PAR accuracy” the authors have made improvements to the text to qualify this area of their analysis. They should go further by adding at the end of the sentence commencing at Line 272 a further clause such as “...although this would need to be tested in appropriately designed future studies”**

Response) Thank you for your careful reading. We have added the phrase as follows (Lines 274–277):

*“Although the small number of nurses makes statistical comparison impossible, it seems that sufficient work experience as an RRT nurse may be an important component of a nurse’s ability to correctly predict patient prognosis; this needs to be tested in appropriately designed studies in the future.”*

**Reviewer #3**

**1. I think that the authors have de-emphasized the experience factor, however, see comments above. If you truly de-emphasize the splitting of one nurse, your study conclusion changes = PAR+ViEWS is superior to PAR.**

Response) We understood your concern, and the authors undertook deep discussion with this issue. With help of a qualified statistician from Medical Research Collaborating Center of Seoul National University Bundang Hospital, we agreed that AUROC is likely to rise with addition of other factors within the same group. Therefore, PAR+ViEWS is highly likely to have larger AUROC in predicting the composite outcome, compared to PAR or ViEWS alone, when compared within the same population. This problem could be solved by dividing the population into two: derivation cohort and validation cohort. Logistic regression model should be derived from the derivation cohort, and the AUROC must be calculated within the validation cohort.

With all due respect to the reviewer, however, comparison of PAR+ViEWS against PAR was not the main focus of our study. We aimed to validate the performance of PAR, an already existing subjective scoring system, by comparing it against well-known objective scoring systems. Introduction of PAR+ViEWS was to carefully suggest a method to back-up the innate flaws of PAR: its subjective nature. Inexperienced nurses should be supervised, and well-known scoring systems could be utilized for this purpose.

We agree that de-emphasizing the experience of nurses could mislead the readers. Sentences were amended as follows (Lines 215–219, 271–277, and 306–310):

*“**To compensate for the smaller AUROC of less-experienced nurses’ PAR, we utilized a logistic regression model of PAR with the warning score of the largest AUROC in our study: ViEWS. In the overall population, the combined model of PAR and ViEWS showed improved AUROC (0.875 [95% CI, 0.849–0.900]) compared to that of PAR alone (0.868 [95% CI 0.843–0.894]); however, this should be interpreted with caution due to the lack of separation of derivation and validation groups.”*

*“One of the major drawbacks of PAR is its subjective nature. As shown in our study,* *the reliability of PAR was questionable: PAR of nurses with longer experience in the RRT revealed an AUROC of over 0.9 in predicting mortality and/or ICU admission within the next day, but those with less experience (<6 months to 1 year) showed an AUROC of under 0.8.* *Although the small number of nurses makes statistical comparison impossible, it seems that sufficient work experience as an RRT nurse may be an important component of a nurse’s ability to correctly predict patient prognosis; this needs to be tested in appropriately designed studies in the future.”*

*“****Conclusions***

*In conclusion, subjective assessment of the patient by an experienced RRT nurse, represented as PAR, reveals good performance in predicting patient prognosis. Although early warning scores may be useful for identifying at-risk patients, direct examinations by healthcare professionals should be emphasized when RRT is activated.”*

**2. The PAR+ViEWS is superior to all of these scores?**

Response) Thank you for your idea. Various combinations of scores were compared: PAR+MEWS, PAR+ViEWS, PAR+SEWS, and PAR+CART. The AUROC and 95% CI were as follows: 0.875 (0.849–0.900) for PAR+MEWS, 0.875 (0.850–0.900) for PAR+ViEWS, 0.874 (0.848–0.899) for PAR+SEWS, and 0.873 (0.847–0.898) for PAR+CART. The differences of AUROCs between these combinations were not significant (*P*=0.710).

**3. Not every facility has nurses with a large amount of experience in an RRT team (in fact all of the nurses were quite experienced overall). There is such a small sample size that eliminating one of the nurses because they did not score similar to the others is not possible here. The PAR+ViEWS was superior to the PAR. The risk of n=5 is you have outliers; conversely, your group with a large amount of direct RRT-experienced nurses is probably less like RRT teams around the world – where there could be very inexperienced nurses. Again, this is the risk you run with your trial. The fact that if you take out the inexperienced nurse is a small discussion point, not a major conclusion.**

Response) We deeply understand your concern, and agree that addition of ViEWS to PAR is only a small discussion point, rather than a major conclusion. We answered this issue along with the response to comment 1.

**4. I would also state this in limitations – that you did not collect direct data on the PAR used for final triage by the physician/and or final decision maker.**

Response) We appreciate your comment. The limitation section was amended as follows (Lines 294–298):

*“**This study has several limitations. First, this study had a single-center design and included only 5 RRT nurses with long working experience, and thus, our results may not be generalizable to other healthcare professionals or less experienced nurses. Inter-observer variability should be considered, and further studies on healthcare professionals with various backgrounds can enforce the strength of PAR.”*

**5. Were any further outcomes assessed? This should be mentioned in the limitation section, that we do not have this data and ICU admission as an outcome does not account for inappropriate/unneeded ICU admissions. Really, the outcome that is more important is mortality and functional status.**

Response) Thank you for the comment. A sentence was added to the limitations section as follows (Lines 303–304):

*“Fourth, outcomes other than short-term prognosis, including unnecessary ICU admissions, long-term mortality, and functional status, were not included.”*

**6. The ACGME 80 work limit has been in effect for a very long time. There was a policy to no 24 hour shifts for interns in 2011 that was revoked in the recent guidelines. There is a lot of data on increased hand offs over time in healthcare, don’t go down this pathway as it is confusing. Cite a large healthcare quality or management study about this issue.**

Response) We deeply appreciate your idea. The reference was replaced with a systematic review by West CP and his colleagues, published in Lancet, 2016. The sentence was revised as follows (Lines 248–250):

*“**To prevent and reduce physician burnout, placing limitations on duty hours can be effective [20], which may lead to an increased need for non-physician healthcare professionals.”*
