# Supplementary material for: Performance of patient acuity rating by rapid response team nurses for predicting short-term prognosis
Source: PLoS One. 2019 Nov 14;14(11):e0225229. doi: 10.1371/journal.pone.0225229 (PMC6855430; doi:10.1371/journal.pone.0225229)
Supplement: Attachment — Submitted filename: 1_Response_to_editor_and_reviewer_1030.docx [file pone.0225229.s009.docx]

**Dear editor:**

We would like to thank all the editors and reviewers for providing us with their valuable opinions about our manuscript. We have revised our manuscript according to the comments and recommendations of the reviewers. We have highlighted all changes in the revised manuscript with “Track changes” function, and a clean copy of the manuscript is also uploaded. Below, we have included an itemized series of responses to the comments of the reviewers.

**Reviewer #2:**

**The authors have addressed my comments relating to the previous submission which has improved the manuscript. My only reservation is the part of the manuscript which focuses on the impact of the experience of the participating nurses on the predictive power of PAR scores when there is such a small sample size (n=5 nurses). The manuscript would be improved by removal of these results and related sections of the discussion altogether in order to maintain emphasis on the principal finding.**

Response) We appreciate your comment. After careful discussion by the authors, we have decided to remove the contents associated with the impact of the experience of the participating nurses on the predictive power or PAR. The relevant sentences were deleted throughout the manuscript, along with Table 4.
